# Effect of Euthanasia Education on the Views of Nursing Students

**DOI:** 10.4314/ejhs.v32i5.13

**Published:** 2022-09

**Authors:** Şerife Yilmaz, Gamze Özbek Güven

**Affiliations:** 1 Dr. Duzce University Faculty of Health Sciences, Department of Nursing History and Ethics, Duzce, Türkiye; 2 Assistant Professor Doctor Malatya Turgut Özal University Faculty of Medicine, Department of Medical Ethics and History, Malatya, Türkiye

**Keywords:** Euthanasia, nursing students, ethics awareness

## Abstract

**Background:**

Nurses play an important role in the healthcare team and are closely involved in the daily care of patients. Sometimes, they are involved in the care of terminal patients, who may thus be confronted with difficult end-of-life decisions such as euthanasia. Therefore, it is important to determine the views on euthanasia of nursing students who will work in health care and prepare them for such situations in their professional life. This study aimed to evaluate the effect of euthanasia education on nursing students' opinions.

**Methods:**

Pretest-posttest single group semi-experimental method was used in the study. Data were obtained with two surveys carried out both before and after education. Descriptive statistics, Chi-square test, or Fisher's exact tests were used to analyze.

**Results:**

Euthanasia education provided students with a different perspective. The results indicated that 56.4% of the students that they knew about euthanasia partially enough before education. After the training, 65.5% of them indicated that they had enough knowledge. Rate of those who are undecided on many questions before education decreased.

**Conclusions:**

Education can significantly change nursing students' approach to euthanasia and provides ethical awareness. Euthanasia and related topics should be added to the curriculum. Students should be encouraged to express their opinions and questions about euthanasia. Students should receive ethics education to prepare them to deal with euthanasia-related issues in their professional lives.

## Introduction

Euthanasia is an important issue that is frequently discussed throughout the world with its medical, ethical, social, economic, political, and religious aspects. The origin of the word euthanasia is Greek. “Eu” means good and “thanatosis” means death, and euthanasia means good death. In Turkish, euthanasia is defined as “good and gentle death” and “right to die” (1). The meaning of euthanasia in medicine; is to allow patients who cannot be treated and cannot provide quality life, to end their lives if they wish (2). According to the American Medical Association, euthanasia is the administration of a lethal agent by another person to a patient suffering from a terminal, painful, debilitating illness to relieve the patient's intolerable and incurable suffering (3). In this study, it was used the concept of “euthanasia,” which indicates a broad definition of euthanasia including both active and passive euthanasia. And, the concept of euthanasia which was used in the survey indicates this broad definition.

Euthanasia is a concept that has been discussed in many countries worldwide. It is legal in some countries (e.g., Netherlands, Belgium, Luxemburg, Canada, and Spain). But it is accepted as illegal in most other countries (4,5). In some countries, euthanasia is considered a patient's right. On the other hand, all kinds of euthanasia are considered a crime in many countries. Especially in Muslim countries, euthanasia is not legal. Turkey is a predominantly Muslim country and in Turkey, euthanasia is not legal. It is a crime according to Turkish Criminal Law and the Patients' Rights Directive, and it is accepted that it should be considered willful murder (6,7).

Nurses play an important role in the healthcare team and are closely involved in the daily care of patients. Sometimes, they are involved in the care of terminal patients, who may thus be confronted with difficult end-of-life decisions such as euthanasia. Therefore, it is important to determine the views on euthanasia of nursing students who will work in health care and prepare them for such situations in their professional life (8,9).

It is very important to include topics related to euthanasia and ethical, political and legal dimensions of euthanasia in nursing curricula. It is necessary for students to be ethically aware and to develop appropriate behaviour in situations that they can meet in their professional life. In the literature, there are some studies in which the views of healthcare professionals and students are discussed. Studies on euthanasia in Turkey are generally about the knowledge, perception, view, and attitudes of physicians, nurses or students toward euthanasia and the factors affecting them (10–13). No studies have been found showing how much euthanasia is included in the curriculum. The studies show that nurses and physicians think that education about euthanasia is insufficient (14–18). In this study, the effect of euthanasia education to 3rd-grade students of Health Sciences Faculty Nursing Department within the scope of ethics course on students' opinions was evaluated.

## Material and Methods

The pretest-posttest single group semi-experimental method was used in the study. The data collection form that was created by the researcher to reveal the views of the students about euthanasia and determine demographic characteristics based on the literature was used before and after the education. The form was evaluated for suitability by two ethics lecturers. Redundant items and unnecessary questions were then removed and some items/questions which were more appropriate were added. In this way, the form reached its final version.

**Participants**: The study population consisted of third-year nursing students who participated in ethics courses during the spring semester of the 2020–2021 academic year at Duzce University Faculty of Health Sciences (n=70). No sample selection was made. All students were invited to study. The study was completed with students who declared volunteering and completed the pre-and post-education data collection form completely (n=55). The response rate in the research is 78.57%.

**Data collection**: Before education, a data collection form was used online. After that, students watched “The Sea Inside”. It is a drama film that won the Academy Award for Best Foreign Language Film. It is based on the real-life story of a man, who was left quadriplegic after an accident, and his request for euthanasia and the right to end his life. Movies are used in the education of health professionals as a powerful and effective educational tool that increases learning potential and experience (19,20). So, the movie was watched within the scope of education for students to better understand the subject. Euthanasia education was provided after the movie. The training was carried out online for 4 hours due to the pandemic by an educator who is an expert in the field of ethics. The training content included euthanasia, concepts related to euthanasia, types of euthanasia and ethical arguments for euthanasia. The educator was careful not to be biased about euthanasia. All the arguments related to euthanasia were explained to the students and they were allowed to discuss them. The assumption was that students gain their ethical perspectives and gain the ability to ethically justify their arguments on euthanasia. Two weeks after the training, the same form was administered to the students again online.

**Data analysis**: Data were transferred to and analyzed using IBM's SPSS Statistics 22 software program. The effect of the education on students' views on euthanasia was examined. Descriptive statistics, Chi-square test, or Fisher's exact tests were used to analyze. P-values less than 0.05 were accepted as statistically significant.

**Ethics approval**: Ethical permission for this study was granted by the relevant body (Duzce University Faculty of Medicine Ethics Committee for Invasive Non-Invasive Clinical Research); permission from the institution in which the study was carried out was also granted. All student participants who attended the training were informed and verbal consent was obtained.

## Results

Of the participants, 83.6% were female and 16.4% were male. Their mean age was 20.75 ± 1.14 years; 29.1% of them were Health Vocational High School graduates; 41.8 % lived in a metropolis; most of their parents were primary or secondary school graduates; the economic situation of 83.6% was middle-income level; 92.7% stated they had received no training course/education on the concept of euthanasia; 60.0% stated that they did not witness the death of a relative; 70.9% stated that they did not witness any patient's death. The demographic characteristics of the participants are presented in Table 1.

**Table 1:** Summary of the demographic characteristics of participants (n=55)

Variable	Number	%
**Gender**		
Female	46	83.6
Male	9	16.4
**School Graduated**		
Health Vocational High School	16	29.1
Other high schools	39	70.9
**Longest-lived place**		
Metropolitan	23	41.8
Urban	24	43.6
Rural	8	14.6
**The educational level of the mother**		
Primary school	32	58.2
Secondary school	8	14.5
High school	8	14.5
University	2	3.6
Illiterate	5	9.1
**The educational level of the father**		
Primary school	18	32.7
Secondary school	18	32.7
High school	13	23.6
University	6	10.9
**Income Level**		
High	7	12.7
Low	2	3.6
Medium	46	83.6
**Had Euthanasia Education?**		
Yes	4	7.3
No	51	92.7
**Experience of Witnessing The Death of Any Relative**		
Yes	22	40.0
No	33	60.0
**Experience of Witnessing The Death of Any Patient**		
Yes	16	29.1
No	39	70.9
**Total**	**55**	**100**

Data were obtained with two surveys carried out both before and after education. The results were summarized in Table 2. Of the students, 56.4% reported that they knew about euthanasia partially enough before education. After the training, 65.5% of them indicated that they had enough knowledge. These results show a statistically significant difference (p=0.000).

**Table 2:** Nursing students' views on euthanasia obtained before and after the training (n=55)

Variables	Before Education		After Education		p-value

	n	%	n	%	
**The level of knowledge about euthanasia**					
Somewhat sufficient	31	56.4	18	32.7	0.000
Sufficient	2	3.6	36	65.5	
Insufficient	22	40.0	1	1.8	
**Euthanasia is ethically and morally acceptable**					
Yes	16	29.1	29	52.7	0.014
No	7	12.7	9	16.4	
Indecisive	32	58.2	17	30.9	
**If you were diagnosed with a terminal illness, would you like to have the option to end your life instead** **of suffering?**					
Yes	20	36.4	23	41.8	0.451
No	19	34.5	13	23.6	
Indecisive	16	29.1	19	34.5	
**If any of your kinsmen was suffering from a deadly disease, and he/she wanted to end her/his life,** **would you allow her/him?**					
Yes	16	29.1	24	43.6	0.279
No	16	29.1	12	21.8	
Indecisive	23	41.8	19	34.5	
**Would you like to be euthanized for suffering and dying patient?**					
Yes	20	36.4	28	50.9	0.262
No	10	18.2	6	10.9	
Indecisive	25	45.5	21	38.2	
**Should a patient who wants euthanasia be euthanized?**					
Yes	32	58.2	33	60.0	0.374
No	5	9.1	9	16.4	
Indecisive	18	32.7	13	23.6	
**Should euthanasia be a right?**					
Yes	30	54.5	32	58.2	0.322
No	5	9.1	9	16.4	
Indecisive	20	36.4	14	25.5	
**Should people have the right to end their life?**					
Yes	28	50.9	28	50.9	1.000
No	12	21.8	12	21.8	
Indecisive	15	27.3	15	27.3	
**Who should have priority in making euthanasia decisions?**					
Patient/relative	55	100.0	53	96.4	0.495
Physician	0	0.0	2	3.6	
**Are religious beliefs a factor that prevents euthanasia?**					
Yes	47	85.5	52	94.5	0.257
No	4	7.3	2	3.6	
Indecisive	4	7.3	1	1.8	
**Do you think euthanasia is applied in your country?**					
Don't know	25	45.5	6	10.9	0.000
I think passive euthanasia is applied	5	9.1	28	50.9	
I think euthanasia is not performed in any condition	25	45.5	21	38.2	
**Do you think euthanasia should be legalized apply in your country?**					
Yes	20	36.4	25	45.5	0.284
No	11	20.0	14	25.5	
Indecisive	24	43.6	16	29.1	
**If euthanasia is legal, would you like to take part in the team administering euthanasia?**					
Yes	4	7.3	3	5.5	0.045
No	35	63.6	46	83.6	
Indecisive	16	29.1	6	10.9	
**Who should administer euthanasia?**					
A professional person trained in euthanasia	46	83.6	51	92.7	0.215
The patient himself/herself	7	12.7	4	7.3	
Physician/Nurse	2	3.6	0	0.0	
**If euthanasia is legal, to whom should it be applied?** *					
Patients with brain death	38	69.1	31	58.5	0.010
Patients with poor prognosis and suffering pain	37	67.3	41	77.4	
Patients on life support	15	27.3	27	50.9	
Terminally ill	18	32.7	24	45.3	
Patients who bedridden, unable to care for themselves	6	10.9	13	24.5	
All patients who want to end their life	14	25.5	21	39.6	
**What do you think about euthanasia?** *					
Euthanasia is the desire to end a person's own life	47	85.5	44	83.0	0.001
Euthanasia is suicide	11	20.0	3	5.7	
Euthanasia is murder and willful murder	7	12.7	1	1.9	
Euthanasia is opposing god, nature, cosmos	9	16.4	7	13.2	
Euthanasia is a medical practice	16	29.1	30	56.6	
Euthanasia is a human-patient right	22	40.0	32	60.4	
Euthanasia is a conscientious responsibility	15	27.3	19	35.8	

* Percentages and totals are based on respondents

Before the education, 29.1% of the students stated that euthanasia was ethical; 58.2% of them stated that they are undecided that euthanasia ethical. After the training, 52.7% thought that euthanasia was ethical, and 30.9% were indecisive. The difference was found to be statistically significant (p=0.014).

There was a statistical difference between the students' answers to the question “Do you think euthanasia is applied in your country?” pre-and post-education. After the training, the rate of “I don't know” response decreased, and the rate of “ I think passive euthanasia is applied” response increased.

Most of the students expressed that they would not want to take an active role in euthanasia both before and after training. Before the training, 29.1% of the students were undecided about this issue, after the education 10.9% of them were undecided. This was found to be statistically significant (p=0.045).

The difference between the students' opinions before and after the education about who would be administered euthanasia if it were legalized was found to be statistically significant (p=0.010). In the same way, the difference in students' opinions about euthanasia before and after the education was found to be statistically significant (p=0.001). After the training, it was determined that the rate of those who said: “euthanasia is a suicide, murder, and willful murder” decreased and the rate of those who said; “euthanasia is a medical practice, a human right” increased.

Most of the students expressed that religious beliefs may prevent euthanasia both before and after training.

Additionally, although not statistically significant, students' indecisive answers generally decreased after the training. Based on this, it can be said that education has provided students to clarify their opinions. The indecisive response rate of the questions “Euthanasia is ethically and morally acceptable”, “If any of your kinsmen was suffering from a deadly disease, and he/she wanted to end her/his life, would you allow her/him?”, “ If any of your kinsmen was suffering from a deadly disease, and he/she wanted to end her/his life, would you allow her/him?”, “ Should a patient who wants euthanasia be euthanized?”, “Should euthanasia be a right?”, and “Do you think euthanasia should be legalized in your country?” were decreased. It can be said that education has decreased the question marks in their minds on some issues.

## Discussion

Euthanasia is a multidimensional subject that has been often debated in health ethics and many factors affect attitudes and decisions regarding euthanasia (21). Knowledge guides attitudes and behaviours. It is necessary to know about the subject to express an opinion on a subject, to be able to comment and to form an attitude. Knowledge is important, especially regarding complex issues such as euthanasia. Nurses are the only health care professionals that provide 24 hours bedside care. They have a substantial role in providing end-of-life care to patients and their families (22). The euthanasia decision is very difficult for patients, their relatives, and healthcare professionals.

Nurses work in close contact with patients and are primarily responsible for their care. So they may face euthanasia decisions. Therefore, nurses need a sound theoretical knowledge of ethical principles to be able to form opinions on issues such as euthanasia (21). So, is important that nursing students are adequately prepared for issues that they will encounter in their professional life before graduation (21,22). Students are educated about euthanasia in their education life, their awareness is provided, and they should be at a level to be able to provide counselling to families. For this reason, euthanasia and similar conflict subjects should be included in the curriculum. Schwartz et al. (2005) reported that students who received end-of-life education showed less concern regarding working with dying patients than those without such education (23). In a Turkish study of 600 nursing students, nearly a third (32.4%) were against euthanasia. Interestingly the majority (78.9%) indicated that they had not received any education on the topic and less than half (42.5%) felt that this would be useful (6). Nursing students participating in Dennis Demedts' study stated that euthanasia had not been a topic during their education (61.9%) (8). Iglesias et al. 2011 and Leuter et al. 2013 also advocate training for nursing students about euthanasia (24,25). We also think that the subject of euthanasia should be included in the nursing curriculum. We found that students' opinions changed over after education. After the training, the rate of students who found their knowledge of euthanasia sufficient increased statistically significantly. Also, there was a significant increase in the number of students who think euthanasia is ethical.

Discussions on euthanasia are mainly carried out within the context of a human right to life, autonomy, and health care professionals' responsibilities. An opinion about euthanasia is as follows; euthanasia is against the basic philosophy of medicine that “human life should be respected in any condition”. It reduces people's confidence in medicine. If active euthanasia is legalized, people will lose confidence in the medical profession’. The other opinion is if there is a “right to life”, “right to death” should also exist. Studies showed that students have different views on this issue (5,26–28). Some studies showed that most students do not want euthanasia for themselves, their relatives, or a terminal patient who is suffering (9,16,18,27,29–33). Similar results were found in our study. It can be said that education provides students with a different perspective. Education is important to provide that students are aware of ethical discussions on euthanasia and to enable them to express their opinions. There are studies showing how much the subject of euthanasia is included in nursing curricula that have not been found in Turkey. Studies on euthanasia are generally related to the knowledge, perception, perspective, and attitudes of physicians, nurses or students toward euthanasia and the factors affecting them (10–13). Therefore, training should be organized to enable different disciplines such as physicians, nurses and midwifery students to create their arguments.

Opinions on euthanasia are affected by many factors. One of the important factors affecting the decision to euthanasia is religious beliefs. Religion plays a key role in euthanasia attitude and perspective (34–36). The beliefs of Islam, Christianity, and Judaism do not allow euthanasia, according to some people belonging to these religions, human life is very important; it is sacred, and should be treated with the highest value. So euthanasia is forbidden. But the people who believe that everything is limited in this world and there is no life after death can apply voluntary euthanasia (37–39). The higher the level of religiosity, the more people have a significantly negative attitude toward euthanasia compared with others (4,40). Studies have shown that most students think that religious beliefs may prevent euthanasia (16,18,31,41–43). Similarly, students in our study said that religious beliefs were affected both before and after the education.

Another factor affecting the decision of euthanasia is the legal rules of the country of residence. Australian students who participated in the study of Adesina et al. (2014) specifically described concerns about legal and moral conflicts between law and ethics concerning euthanasia (22). Similarly, a study from Poland stated that nearly half of the nursing students (49.6%) were opposed to active euthanasia, but favoured the legalisation of euthanasia (43).

Students have different opinions on the legalization of euthanasia. Some studies showed that students opposed the legalization of euthanasia (16,18,29). In other studies, students supported the legalization of euthanasia (33,43). In addition, it has been observed that most students don't want to take part in the application of euthanasia if it were legal (16,18,33). The results are similar to our study.

In addition, similar to the literature, the students who participated think that euthanasia should be applied by a professional person trained in euthanasia (16,18).

Students answered the question of “Whom should euthanasia be applied to in case of legalization”, the patients with the highest rate of brain death before and after the education, and the patients who suffered severe pain with a poor prognosis. Similarly, in the studies of Cetinkaya and Karabulut 2016 and Karaarslan et al., the most common answers were patients with brain death, patients with severe pain, and the terminally ill (16,44).

There was a significant difference between pre-and post-training about thoughts on euthanasia practice. The percentage of students who marked euthanasia as suicide, murder and deliberate homicide before the training decreased after the training. But the percentage of those who marked euthanasia as a medical practice with a humanpatient right, the proportion of students marking conscientious responsibility increased after the education. In the other studies, most of the students often defined euthanasia as going against god/nature/creator/cosmos, murder, and voluntary manslaughter (16,18). Based on this, it can be said that education provides students with a different perspective on euthanasia.

Teaching euthanasia and related concepts in education will clear up the confusion of students and contribute to their clear views on euthanasia. Before the education, students could not express their opinions because they did not know the difference between definitions such as active euthanasia, passive euthanasia, and assisted suicide. It is thought that students can express their opinions more easily after learning these definitions. In addition, in the study, we thought that the reason for a shift towards a more pro-euthanasia stance among the nursing students was that they learned the definitions. Since the students learned the definitions clearly, they were able to express their opinions more clearly. The percentage of students who marked euthanasia as suicide, murder and deliberate homicide before the training decreased after the training. We thought that this was because students learned the difference between definitions.

In summary; it can be said that euthanasia education provides students with a different perspective, the rate of those who are undecided on many questions before education decreases, and it enables them to think about subjects that they may not have thought about before with education. These findings suggest that education can significantly change a nursing student's approach to euthanasia, and provides ethics awareness. Euthanasia and related topics should be added to the curriculum. Students should be encouraged to express their opinions and questions about euthanasia. Students should receive ethics education to prepare them to deal with euthanasia-related issues in their professional lives. In addition, the scope of the study should be expanded by conducting similar studies in medical education.
